# Resolving the Enigma of the Clonal Expansion of mtDNA Deletions

**DOI:** 10.3390/genes9030126

**Published:** 2018-02-27

**Authors:** Axel Kowald, Thomas B.L. Kirkwood

**Affiliations:** 1 Institute of Cell and Molecular Biosciences and Institute for Ageing, Campus for Ageing and Vitality, Newcastle University, Newcastle upon Tyne NE4 5PL, UK; tom.kirkwood@ncl.ac.uk; 2 Center for Healthy Aging, Department of Cellular and Molecular Medicine, University of Copenhagen, Copenhagen 2200, Denmark

**Keywords:** aging, mitochondrial deletion mutants, model of clonal expansion

## Abstract

Mitochondria are cell organelles that are special since they contain their own genetic material in the form of mitochondrial DNA (mtDNA). Damage and mutations of mtDNA are not only involved in several inherited human diseases but are also widely thought to play an important role during aging. In both cases, point mutations or large deletions accumulate inside cells, leading to functional impairment once a certain threshold has been surpassed. In most cases, it is a single type of mutant that clonally expands and out-competes the wild type mtDNA, with different mutant molecules being amplified in different cells. The challenge is to explain where the selection advantage for the accumulation comes from, why such a large range of different deletions seem to possess this advantage, and how this process can scale to species with different lifespans such as those of rats and man. From this perspective, we provide an overview of current ideas, present an update of our own proposal, and discuss the wider relevance of the phenomenon for aging.

## 1. Introduction

Aging involves a gradual decline of an organism’s functional status, leading to an increasing risk of death with chronological age. In view of the growing life expectancy of modern societies [1] and the strain it places on existing health care and pension systems, it becomes ever more important to understand the biochemical mechanisms of this deteriorating process. An intriguing hallmark of aging in mammals is the appearance of cells carrying significant burdens of mitochondrial DNA (mtDNA) mutants [2,3,4,5,6,7,8]. Unlike the mtDNA mutations which cause inherited diseases, those associated with aging appear to be somatically acquired. Within a given tissue, there is often considerable heterogeneity in the burden of mtDNA mutations, such that affected cells co-exist side by side with healthy cells that carry few, if any, mutations. Furthermore, the frequency of affected cells tends to increase with age and there is evidence that within individual cells, the mitochondrial population is commonly overtaken by a single mutant type, very often a deletion in which a part of the normal mtDNA genome has been lost. The precise mutations tend to differ from one affected cell to another, suggesting that individual mtDNA mutations arise at random. How these mtDNA mutations undergo clonal expansion is a question of longstanding interest. The possibilities that they multiply either because of a so-called *vicious cycle* such that defective mitochondria simply generate more reactive oxygen species (ROS), which in turn cause more mutations, or because of random drift, have both been considered but found to be unsatisfactory [9,10]. Instead, it seems most likely that new mtDNA mutations are acted upon by some form of intracellular selection, causing the expansion of a clone of mutant mitochondria that may come to dominate or entirely exclude the wild type population. Understanding the nature of the selection force that drives such clonal expansion is therefore an important challenge in addressing the causes and consequences of mtDNA mutations within the context of the aging of the animal as a whole.

The role of selection in affecting how the frequency of mtDNA deletions changes over time will be influenced by several factors [11]. Firstly, there are typically thousands of mtDNA molecules per cell. Secondly, these mtDNA molecules are distributed among many mitochondria, which undergo processes of duplication and removal that operate independently of cellular divisions. Thirdly, the mitochondria themselves are dynamic organelles that undergo frequent cycles of fusion and fission. Collectively, these factors create a complex evolutionary context wherein any selective advantage or disadvantage of individual mtDNA mutations, operating over rapid timescales, can produce interesting results.

Among the various possibilities to account for a selective advantage favoring mtDNA deletions are that: (i) in a cell where wild type and deleted mtDNA molecules co-exist, there may be a selection advantage for deletion mutants since they have a smaller genome size, which might result in a shorter replication time [12,13,14]; (ii) if mitochondria that are compromised by a high burden of mutations have a slower rate of metabolism, they may be less damaged by ROS and so relatively spared from deletion by mitophagy, thereby resulting in survival-based selection through a process that has been termed *survival of the slowest* [15]; (iii) the selection advantage of mtDNA deletions might be based on features relating to some aspect of the machinery for mtDNA replication, of which several possibilities exist, at least hypothetically [11]. Whichever mechanism might be at work, it is essential that it can be shown to work quantitatively, since the process of selection is in essence a numbers game. Possibility (i) has been closely examined but found to be implausible [16], chiefly because the time required for replication of an mtDNA molecule is only a tiny fraction (<1%) of the half-life of mtDNA, which drastically diminishes any scope for a size-based replication advantage to be important. Possibility (ii) has also been found to be unlikely, since not only is it incompatible with mitochondrial dynamics [17], but it also appears that dysfunctional mitochondria are degraded preferentially rather than more slowly than intact ones [18,19,20,21]. 

**Figure 1:**
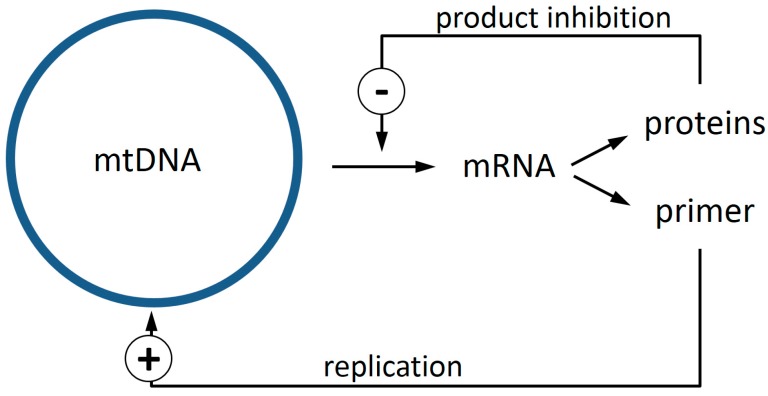
The replication of mitochondrial DNA (mtDNA) in vertebrates is primed via processed mRNA molecules [22,23]. We propose that a product inhibition exists that diminishes transcription (−), and thus replication, if sufficient proteins are available. If a deletion event eliminates the genes for proteins that participate in this feedback, transcription and also replication will not be downregulated in such mutants, resulting in a replication advantage (+).

By a process of elimination, it appears probable, therefore, that the enigma of clonal expansion of mtDNA deletions requires explanation in terms of the machinery for DNA replication. Recently, we noticed that when the locations of mtDNA deletions, which had been reported from rats, rhesus monkeys, and humans, were compared, there was a stretch of mtDNA that was overlapped in nearly every instance [10]. Based on this observation and noting that the primer required for DNA replication is provided by processing an mRNA transcript, we suggested a novel mechanism based on this intimate connection of transcription and replication in mitochondria. If a product inhibition mechanism exists that downregulates the transcription rate if sufficient components for the respiration chain exist, then deletion events removing a region of the genome involved in this feedback-loop would confer to such deletion mutants a higher rate of replication priming, leading to a substantial selection advantage (Figure 1). In this article, we report additional data from mice that are strongly consistent with our previous analysis of rats, monkeys, and humans, and we further examine the implications of the hypothesis that a shared sequence, falling within the common overlap of these many individual deletions, might throw light on the underlying mechanism for clonal expansion.

## 2. Materials and Methods

### 2.1. Computer Simulations of mtDNA Accumulation

Deterministic simulations of the competition between wild type and mutant mtDNA (as shown in Figure 4) were performed by solving the system of differential equations specified in [10]. For this purpose, we used the software tool Mathematica [24]. To compute the effects of the competition between wild type and mutant mtDNA at the tissue level (as shown in Figure 5), we developed a Java program that iterates through the mitochondrial lifecycle in steps of one hour, stochastically performing degradation, replication, mutation, and ATP production according to the rules laid out by the system of differential equations (program available under petition). 

### 2.2. Databases

**Figure 2:**
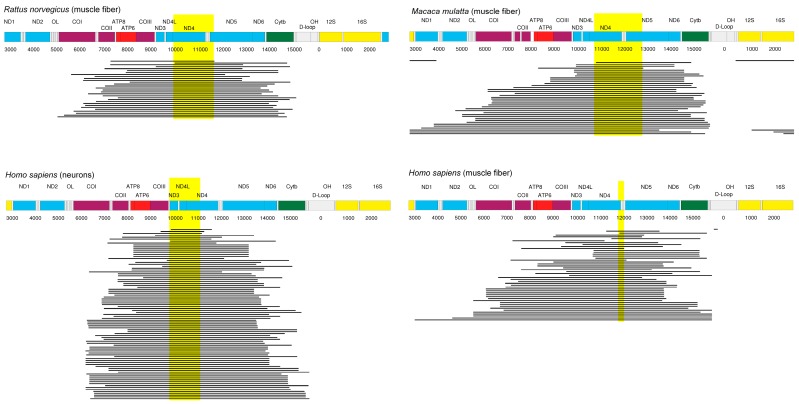
Locations of mtDNA deletions found in rats [2] (top left), rhesus monkeys [3] (top right), and humans [27,28] (bottom). The highlighted area indicates a stretch of mtDNA that is common to all 30 deletions in rat, 38 of 39 deletions in rhesus monkey, all 89 deletions in human neurons (left bottom), and 46 of 48 deletions in human muscle (right bottom). Redrawn from [10].

**Figure 3:**
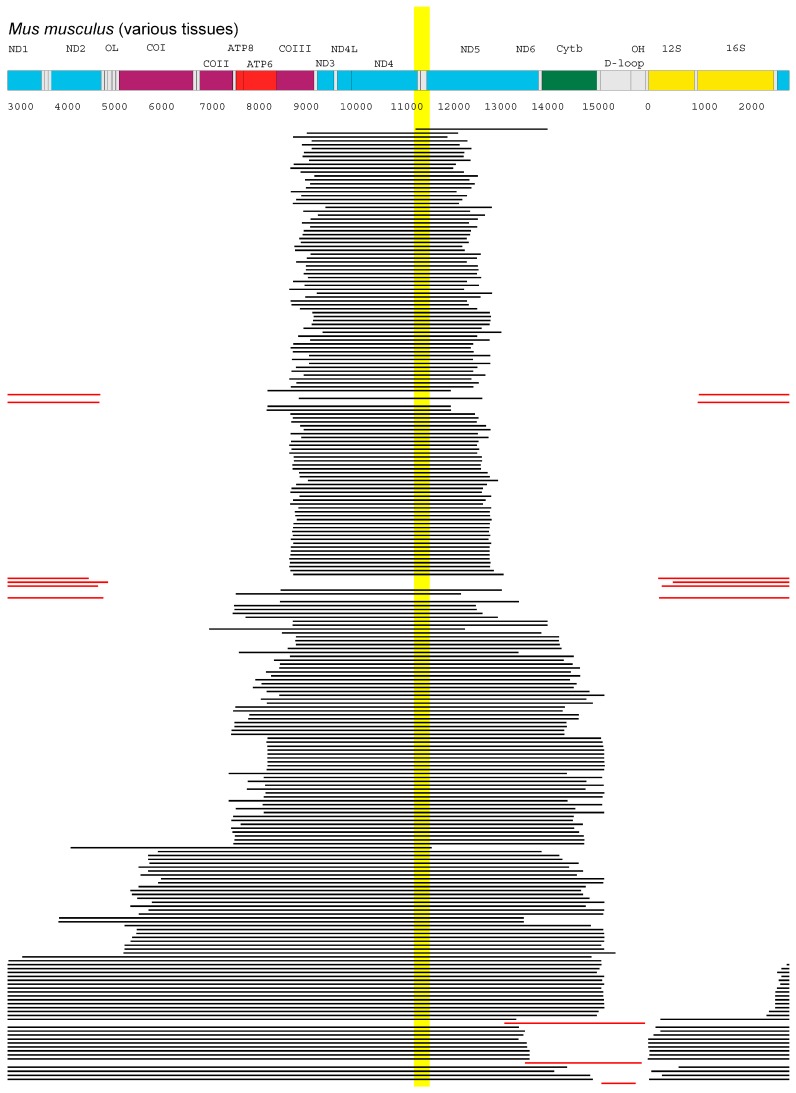
Deletion spectrum of mtDNA deletions found in mice, taken from the MitoBreak database [25]. The data come from 24 publications that studied various tissues using different techniques. The highlighted area indicates a stretch of mtDNA deleted in a total of 236 of the 245 mice samples around the nicotinamide adenine dinucleotide dehydrogenase (NADH) subunit 4 gene (*ND4*) and *ND5* genes. The remaining nine deletions are shown in red.

The mtDNA maps shown in Figure 2 and Figure 3 are based on GenBank entries X14848 (rat), AY612638 (rhesus monkey), 251831106 (human), and 7770098 (mouse). The deletion data shown in Figure 3 were obtained from the MitoBreak database [25].

## 3. Results

DNA polymerases cannot start DNA synthesis *de novo*, but instead require an RNA primer that provides a 3’ end. For nuclear DNA, such an RNA primer is generated by a specialized enzyme, a primase. In vertebrate mitochondria, however, the situation is different. For these organelles, it is known that the primer for heavy strand replication is manufactured by processing a large poly-cistronic mRNA, while it is still attached to the mtDNA [22,23]. This establishes a close connection between transcription and replication and we therefore assume (like others [22]) that the transcription rate and the level of replication initiation exhibit a positive correlation in mitochondria.

To explain the accumulation of mitochondrial deletion mutants, we furthermore proposed that there is a simple feedback inhibition that downregulates transcription if enough products (proteins) are present (Figure 1). Such a system runs into problems, if genes that are involved in this feedback are removed from the mtDNA. Those deletion mutants are no longer capable of downregulating their transcription rate and consequently also have a higher rate of replication priming. In principle, such deletion mutants cannot synthesize all the proteins that are required for a functional respiratory chain. This should lead to a severe energy shortage and concomitantly result in a growth deficit compared to wild type mtDNA. But recent years have shown that mitochondria are not the distinct entities that can be seen in electron micrographs. Instead, it is now clear that they form a very dynamic network through constant and rapid fission and fusion processes [18,26]. This allows the deletion mutants to parasitize on the ATP pool created by wild type mtDNA and out-compete them via the increased replication rate. Thus, instead of being a rescue mechanism, fusion processes seem to open the door for the spread of selfish mtDNA mutants. An evolutionary interpretation of why fusion is nevertheless required has recently been proposed [17]. 

It is *a priori* not possible to predict which gene products should be involved in the feedback mechanism, but the analysis of experimental data on clonally amplified mtDNA deletions provided important clues [10]. Based on the investigation of single cell studies in rats [2], rhesus monkeys [3], and humans [27,28], it became clear that the overwhelming majority of mtDNA deletions have as a common region the nicotinamide adenine dinucleotide dehydrogenase (NADH) subunit 4 gene (*ND4*), which is part of complex I of the respiration chain (Figure 2).

Thus, the deletion spectra are most easily explained if the *ND4* and maybe *ND5* gene would be involved in the hypothetical negative feedback mechanism that is supposed to control mitochondrial transcription. The data from Figure 2 stem from single cell studies that were performed in elderly, healthy individuals. To corroborate the findings, it would be interesting to add and investigate deletion data for further species. We therefore looked at the MitoBreak database [25] that contains 245 mtDNA deletions found in mice, collected from 24 publications. Although the data come from a mixture of publications, not all of which involve healthy individuals or are based on single cell experiments, it is remarkable to see that also in this data set 96.3% of the deletions have an mtDNA region in common and that this region again affects the *ND4* and *ND5* genes, as seen in Figure 3. 

While the experimental deletion data support the idea of a feedback mechanism, it is also important to know if the timeframe of the proposed expansion mechanism is feasible. For this purpose, we developed a small mathematical model that describes the synthesis and degradation of mutant and wild type mtDNAs that are controlled via the cellular demand for energy [10]. For that model, we assume that differences in transcription rates are proportionally translated into differences in replication rates. 

**Figure 4:**
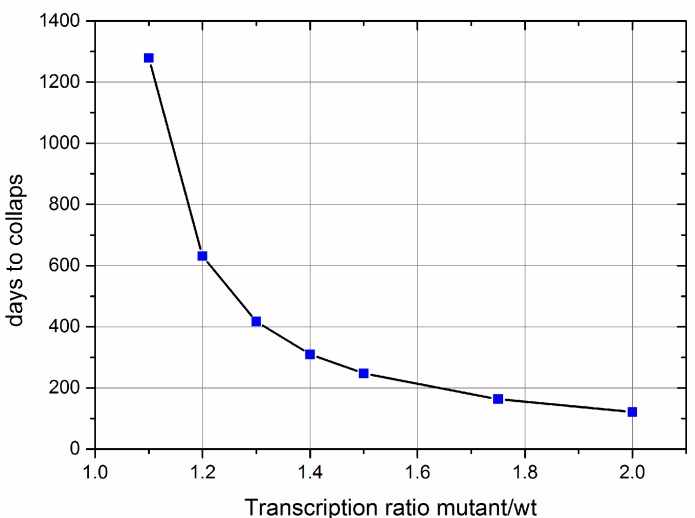
Results of computer simulations of the proposed feedback mechanism for the accumulation of mtDNA deletion mutants. It shows the timespan required from the occurrence of the deletion mutant until the complete takeover of the mitochondrial population depending on the ratio of the transcription rates. Wild type; wt.

Once a deletion mutant occurs in a wild type population of mtDNAs, its fate depends on whether the deletion affects a feedback gene or not. If not, it can only accumulate via random drift and it has been shown that such a mechanism is not capable of explaining the clonal expansion of mtDNA mutants in short lived animals like mice or rats [9]. If, however, the deletion removes a feedback gene, this leads to a higher transcription and therefore replication rate of the mutant mtDNA. It is difficult to predict how large the difference in the transcription rate might be, but Figure 4 shows simulation results that range from 10% to 100% increased transcription in the mutant. This advantage leads to an accumulation of the mutant mtDNA until the cellular requirement for energy can no longer be satisfied, leading to a collapse caused by ATP exhaustion. The timespan of this accumulation process, from first occurrence of the mutant until the complete takeover turns out to be remarkably short, compared with a human lifespan. With a 10% difference in transcription 3.5 years are required, with a 50% difference this time is reduced to eight months, and if there would be a twofold difference in transcription rates, the accumulation is completed within four months. 

Figure 4 describes the accumulation process within a single cell after the deletion occurred. But there are also characteristic hallmarks at the tissue level that happen during the aging process. Old muscle tissues display a mosaic pattern of cytochrome c oxidase (COX)-negative cells, which is caused by the accumulation of mtDNA deletion mutants [6]. Examination of these cells revealed that they display a very low level of heteroplasmy [2,3], which means that in most cases, there is only a single type of mutant mtDNA that has accumulated in the cells. Furthermore, this pattern has been observed in species covering a 30-fold difference in lifespans (e.g., rats vs. human), which represents an important test for hypotheses that aim to explain this phenomenon.

**Figure 5:**
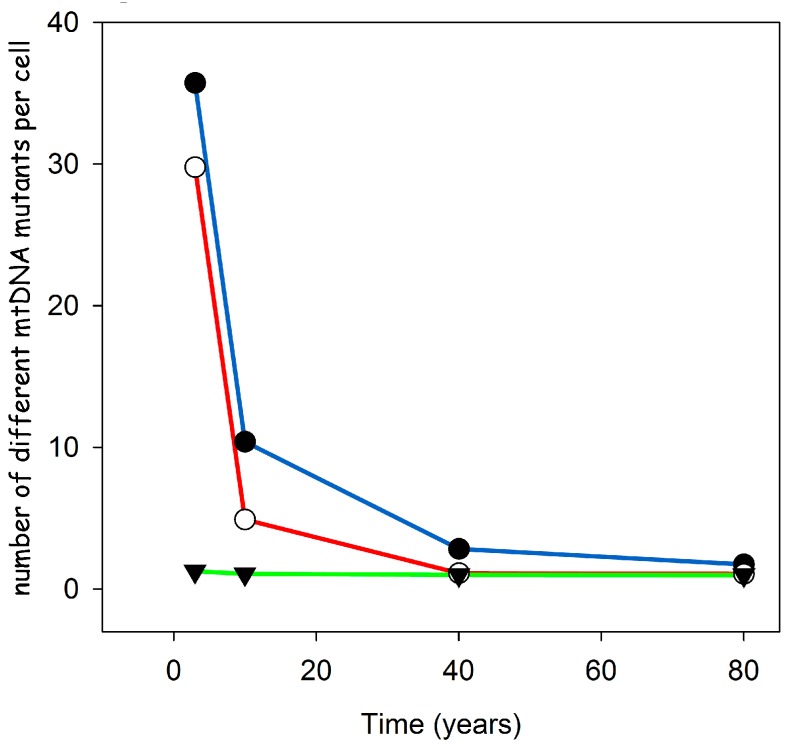
Results of stochastic computer simulations for different species lifespans and different hypotheses (filled circles: random drift, open circles: smaller genome size, filled triangles: replication via transcription) explaining the accumulation of deletion mutants. Only the idea that replication is controlled via a transcriptional feedback leads to the low level of heteroplasmy observed in short lived species. Modified from [10].

To investigate if the proposed feedback mechanism is in agreement with these observations, we performed stochastic computer simulations that started with a wild type population of mtDNAs and allowed for a certain mutation frequency during each replication step [10]. We performed these simulations for various lifespans ranging from three to 80 years, while adjusting the mutation rate such that at the end, ten percent of the cells where taken over by mutant mtDNAs. As can be seen from Figure 5, our proposed mechanism leads to a very low level of heteroplasmy over the whole range of lifespans. Even for a short lifespan such as three years, we only find around 1.2 different types of mtDNA mutants per COX-negative cell. This is in stark contrast to alternative explanations like random drift [9] or a replication advantage based on a smaller genome size [16], which predict for short lived species a very high level of heteroplasmy.

## 4. Discussion

It has long been thought that damage to the mitochondrial genome could not be of major relevance since a typical cell harbors hundreds of mtDNA molecules. Assuming a constant competition between these mtDNAs, it was reasonable to conclude that there would be a continuous selection against defective mutants. However, the discovery that mitochondrial deletion mutants are involved in human diseases [29,30] showed that this reasoning was wrong. Furthermore, it became clear that the clonal accumulation of mtDNA deletion mutants is also involved in the progressive loss of muscle fibers and the aging process [2,3,6,31,32]. Disappointingly, the molecular mechanism that underlies this accumulation of deleterious mtDNA variants has so far eluded definite identification. 

The finding that mitochondria are dynamic structures that constantly exchange material via fission and fusion [26,33] represents an important step forward, showing that all mitochondria of a cell effectively form a single large mitochondrial compartment. Under those circumstances, the competition between individual mtDNA molecules becomes much more complex, since mutants can parasitize on a common pool of ATP and nutrients. Although this helps to understand why there is not a strong selection against defective mutants, it does not help to explain why they should have a selection advantage. Of course, some explanations like the vicious cycle [34,35] or random drift [36,37] do not require a selection advantage, but the vicious cycle predicts a multitude of different mtDNA mutants per cell (instead of a single clonally expanded mutant) and the same is true for random drift in the case of short lived species [9]. 

The shorter size of deletion mutants naturally invites the idea that the selection advantage may rest in a shorter replication time [12,13]. But since the time needed for replication is much shorter than the half-life of mtDNA, it turns out that for short lived species, only a very high mutation rate and concomitantly an unrealistically high level of heteroplasmy could explain the observed accumulation of deletion mutants [16]. Finally, the *survival of the slowest* hypothesis proposed that mitochondria harboring defective mtDNAs have a longer half-life than wild type specimens before they are degraded [15]. This effectively also confers a selection advantage to mutant mtDNAs. However, subsequent experiments have shown that defective mitochondria are actually degraded more rapidly via mitophagy than wild type specimens [18,20,21], which is contrary to the assumptions of this hypothesis.

The idea of a transcriptional feedback mechanism that influences the replication of mutant mtDNA molecules via the availability of RNA primers has none of these problems and offers several attractions:

- It provides a biochemical mechanism that confers a selective advantage to defective mtDNA molecules.
- Experimental data sets from mouse, rat, rhesus monkey, and human specimens all point to a region of mtDNA that is shared between most of the deletions. The genes of this region, *ND4* and possibly *ND5*, are prime candidates for components of the proposed feedback mechanism.
- Computer calculations show that the suggested mechanism leads to a very low level of heteroplasmy, as observed experimentally.
- Importantly, this low level of heteroplasmy is also predicted for short lived animals like mice and rats. This is in stark contrast to other ideas like random drift or size advantage.

Another interesting consequence of our proposal is that the actual size of the deletion should not be relevant for the resulting selection advantage. Any deletion that disables the feedback inhibition will benefit from the same degree of increased transcription rate and replication initiation. A recent publication of Campbell et al. [38] studied single human skeletal muscle fibers and investigated the correlation between the size of the mtDNA deletion and the length of the COX-negative area of the affected muscle fiber. No such correlation was found, indicating that small and large mtDNA deletions have the same selection advantage against wild type molecules, in agreement with the proposed feedback inhibition. Similarly, in *Caenorhabditis elegans* populations, heteroplasmy exists for small and large mtDNA deletions and recent experimental results indicate that the level of mutant mtDNA is independent of the deletion size [39]. However, another study generated transgenic mice with an inducible, mitochondria targeted restriction enzyme and found evidence that large deletions accumulate faster than small ones [40]. These are very different approaches and more work in this direction is needed to settle the question of whether the selection advantage is size dependent.

A further important prediction of our model is that the timespan between the first appearance of a mutant mtDNA until the moment when the cell is completely taken over by the clonally expanded deletion mutant, is rather short. Although it is difficult to predict by how much transcription and thus replication will be increased if the feedback inhibition is impaired, a conservative estimate seems to be somewhere between 10% and 100%. Under those conditions, it takes only a few months to a few years, until the spread of mutant mtDNA is completed (Figure 4). An important factor that enters into these calculations is the half-life of mtDNA. For our computations, we used a value of 10 days, but experimental measurements range from a few days to many months [41,42,43,44]. For further progress, it would therefore be very important to reduce this large uncertainty through state of the art experiments. 

We assume that the timespan necessary for mutant accumulation is the same for different species. Differences in lifespan would not be caused by differences in the transcriptional feedback system, but by differences in the mutation rate that create *de novo* mutant mtDNAs in a wild type population. The short time required for clonal expansion is the key ingredient, which makes it possible that species with a 30-fold difference in life expectancy (from mouse to man) all show an extremely low level of heteroplasmy in affected cells. If such a short accumulation time could be confirmed experimentally, it would therefore represent strong support for our proposed mechanism. A few studies tried to estimate the rate of mutant accumulation and their time of origin [45,46,47] but came to variable results. A feasible approach might involve the transgenic mouse model of Fukui and Moraes [40] mentioned earlier, since it provides precise information about the time of the induction of deletion mutants. Single cell studies at later time points could then reveal information regarding the kinetics of the accumulation.

The simulations of the accumulation of deletion mutants shown in Figure 4 and elsewhere [10] demonstrate that the clonal expansion leads to a gradual decline of the ATP level, which finally leads to the collapse of the system caused by energy exhaustion. Thus, the mathematical model does not allow for a stable heteroplasmy of mutant and wild type mtDNAs. Although such a long-term co-existence of two genotypes has often been observed in human mitochondrial diseases [48,49], it is not clear if such a stable heteroplasmy is also characteristic for the normal age-associated accumulation process. It also seems possible that COX-negative cells finally undergo apoptosis, contributing to the observed age-related appearance of sarcopenia [6,50]. The COX-negative cells found in aged post-mitotic tissues would then only represent a transitional state before their clearance. But in any case, to understand how the cell can maintain a stable heteroplasmy (as in the case of diseases), it will most likely be necessary to look at the mitochondrial quality control mechanisms. It is known that mitophagy is selective in that mitochondria with an impaired membrane potential are both degraded faster and also have a slower rate of fusing with the remaining network [18,19,21,51]. Interestingly, a study investigating the maintenance of mtDNA heteroplasmy in *C. elegans* populations [39] found that there is an intriguing connection between the unfolded protein response (UPR) that is elicited by deletion mutants and the cellular mitophagy system. An active UPR seemingly leads to higher levels of mutant mtDNA by protecting the mutant genome from mitophagy. Clearly, now that a good model is available for the mechanism that confers a selective advantage to deletion mutants, future work will have to concentrate on integrating further cellular mechanisms that control levels of heteroplasmy (like mitophagy and UPR) in the overall picture.

Knowing how the accumulation process of mtDNA deletion in mutants works is important for understanding the problem. But the ultimate aim has to be to cure mitochondrial diseases and prevent the age-related rise of COX-negative cells in organisms. In principle, three different approaches are conceivable. It has been proposed that an important source for deletion mutations is slippage replication at perfect and imperfect direct repeats [52,53,54]. Thus, if mtDNA could be engineered with a diminished level of such repeats, it might be possible to postpone the age-related rise of COX-negative cells, which should effectively delay the aging process. However, such an approach might be feasible for animal models, but not for humans. The second approach is free of such ethical problems and aims to reduce the load of mtDNA mutants inside a single cell. Models of mitophagy-based quality control have shown that, in general, it holds that a more rapid fission and fusion cycle and a higher specificity of degradation lead to a stronger clearance of mtDNA mutants [55]. Furthermore, it could also be shown experimentally that stimulating mitophagy using rapamycin reduced the level of mtDNAs carrying a pathogenic Leber hereditary optic neuropathy (LHON) mutation in tissue culture [56]. Similarly, the overexpression of Parkin stimulates mitophagy and was able to reduce the level of deleterious COXI mutations in human cybrid cells and *Drosophila* [20,57]. Thus, the stimulation of mitophagy opens a promising field of research to reduce the cellular level of mutant mtDNAs in vivo. Whether such an approach would be able to completely clear an affected cell from mutated genomes remains to be seen. 

A third and more speculative approach might be to combat mitochondrial mutants at the tissue level. Cells with a large fraction of mtDNA deletion mutants are significantly impaired. Under those circumstances, it should be expected that those cells undergo apoptosis, but it appears they do not. In the case of muscle cells, it might be possible that apoptosis would lead to negative effects since it removes or breaks a whole muscle fiber [6]. Under those conditions it might be better to suppress apoptosis and somehow keep those cells alive. But as in the case of senescent cells, this might only be possible through the export of toxic substances [58]. In this scenario, there would be something like a *mitochondria associated secretory phenotype* analogous to the senescence associated secretory phenotype seen in the case of cellular senescence. This could explain why a small number of COX-negative cells might have an important functional impact on the organism. Given these similarities between mitochondrial damage caused by mutant accumulation and senescent cells, a similar treatment might also be possible, i.e., by discovering compounds that can induce apoptosis specifically in COX-negative cells. Instead of preventing or reversing the clonal expansion of mtDNA mutants, this strategy would simply remove affected cells from the tissue via a form of *mitolytics* for cells that are taken over by runaway mtDNA mutants. Such an approach would not be suitable for mitochondrial diseases where virtually all cells are affected, but in case of the progressive accumulation of cells that are affected during the aging process, this strategy might be applicable.
